# Addition of 5-fluorouracil to docetaxel/cisplatin does not improve survival in locoregionally advanced nasopharyngeal carcinoma

**DOI:** 10.18632/oncotarget.23300

**Published:** 2017-12-14

**Authors:** Wang Fangzheng, Jiang Chuner, Sun Quanquan, Ye Zhimin, Liu Tongxin, Liu Jiping, Masoto Sakamoto, Wu Peng, Shi Kaiyuan, Qin Weifeng, Fu Zhenfu, Jiang Yangming

**Affiliations:** ^1^ Department of Radiation Oncology, Zhejiang Cancer Hospital, Hangzhou 310022, People’s Republic of China; ^2^ Key Laboratory of Radiation Oncology of Zhejiang Province, Hangzhou 310022, People’s Republic of China; ^3^ Department of Breast Surgery, Zhejiang Cancer Hospital, Hangzhou 310022, People’s Republic of China; ^4^ Department of Physics, Zhejiang Cancer Hospital, Hangzhou 310022, People’s Republic of China; ^5^ Department of Radiology, Fukui Red Cross Hospital, Fukui 918-8501, Japan; ^6^ Department of Pathology, Zhejiang Cancer Hospital, Hangzhou 310022, People’s Republic of China; ^7^ Department of Ultrasonography, Zhejiang Cancer Hospital, Hangzhou 310022, People’s Republic of China; ^8^ Department of Digital Earth, Institute of Remote Sensing and Digital Earth, CAS, Beijing 100101, People’s Republic of China

**Keywords:** nasopharyngeal carcinoma, induction chemotherapy, concurrent chemoradiotherapy, intensity-modulated radiotherapy, toxicity

## Abstract

Addition of induction chemotherapy (IC) to concurrent chemoradiotherapy (CCRT) is a potentially effective approach for treating locoregionally advanced nasopharyngeal carcinoma (NPC). In this study, we compared the efficacy and toxicity of IC regimens consisting of docetaxel plus cisplatin with (TPF) or without (TP) 5-fluorouracil followed by CCRT in these patients. Clinical data from 245 propensity score-matched pairs of newly diagnosed non-metastatic NPC patients who received either TPF or TP IC before CCRT were retrospectively reviewed. After a median follow-up of 60 months, 5-year locoregional relapse-free, distant metastasis-free, progression-free, and overall survival rates were 95.6%, 94.7%, 90.4%, and 92.9% in TPF arm patients and 96.7%, 94.2%, 91.7%, and 91.0% in TP arm patients, respectively. There were thus no differences in survival between the two arms. Multivariate analysis revealed that IC regimen was not an independent prognostic factor for any of the survival outcomes. However, patients who received TP experienced lower incidences of grade 3/4 toxicities than those who received TPF. These results indicate that omission of 5-fluorouracil from TPF-based IC did not affect survival outcomes, but was associated with reduced toxicity, in patients with locoregionally advanced NPC.

## INTRODUCTION

The incidence of nasopharyngeal carcinoma (NPC), which ranges between 15 and 50 cases annually per 100,000 people in Southern China, Singapore, and Malaysia, varies with age, ethnicity, and geographical origin [1]. Radiotherapy (RT) is the standard treatment for NPC due to its anatomical location and high radiosensitivity. Approximately 60-70% of all NPC patients have locoregionally advanced NPC at diagnosis [2]. Intensity modulated radiation therapy (IMRT) has improved locoregional control, but doesn't substantially improve survival outcomes or reduce distant failure rates [3, 4]. According to a meta-analysis of randomized studies, combining RT with chemotherapy reduces the risk of mortality by 18% and increases 5-year survival by 4-6% [5]. Concurrent chemoradiotherapy (CCRT) with or without adjuvant chemotherapy improves overall survival and has become the standard treatment for locoregionally advanced NPC despite the associated acute toxicities [6–8]. A previous meta-analysis showed that the addition of induction chemotherapy (IC) to CCRT reduced distant failure in locoregionally advanced NPC patients [9, 10]; another recent meta-analysis confirmed that IC in addition to CCRT improved progression-free survival (PFS) and overall survival (OS) [11]. However, the efficacy of adding IC to CCRT for patients with locoregionally advanced NPC remains controversial [12–14].

The addition of IC with cisplatin and fluorouracil (PF) to CCRT did not improve survival outcomes in patients with locoregionally advanced NPC, although FP has been widely used in first-line IC in these patients [15, 16]. Taxane, a new anticancer drug, in combination with cisplatin either alone (TP) or with 5-fluorouracil (TPF), improved survival in patients with locoregionally advanced head and neck squamous cell cancer [17–19]. Moreover, the addition of IC with TPF or TP to CCRT also improved survival in locoregionally advanced NPC patients [20–24].

It is unclear which IC regimen is optimal for locoregionally advanced NPC patients. In a phase II study comparing the efficacy and toxicities of IC with TPF and TP followed by CCRT in these patients, we found that TP-based IC resulted in similar survival, but fewer grade 3/4 toxicities, than TPF [25]. However, these results were considered preliminary due to the small sample size and short follow-up duration. We therefore performed this retrospective study to compare long-term survival outcomes after the addition of TPF or TP to CCRT in a large sample of locoregionally advanced NPC patients. To avoid the interference from covariates, we used propensity score matching (PSM) methods to establish patient pairs for comparison [26].

## RESULTS

### Patient characteristics

Clinical data were collected and retrospectively reviewed for a total of 650 newly-diagnosed locoregionally advanced NPC patients who were initially treated with IC followed by CCRT. Based on these data, 245 patient pairs were established using PSM. The median age of the selected subjects was 48 years (range, 18-69 years) and the male to female ratio was 2.33:1 (343 and 147, respectively). All patients completed a full course of radical IMRT and received 2-4 cycles of IC plus concurrent cisplatin for 1-2 cycles. Of the paired patients, 223 (45.5%) patients received AC. The median total cisplatin doses in the two arms were 334 mg/m^2^ and 340 mg/m^2^, respectively. There were no statistically significant differences in age, gender, pathology, stage, or treatment factors between the TPF and TP arms. Basic patient characteristics and therapy adherence between the two arms are shown in Table 1.

**Table 1:** Basic demographic and tumor characteristics of 490 locoregionally advanced NPC patients

Characteristic	TPF regimen	TP regimen	χ^2^	p
	N=245	N=245		
Gender			0.622	0.430
Male	176 (71.8%)	167 (68.2%)		
Female	69 (28.2%)	78 (31.8%)		
Age (years)			0.008	0.927
Range	18-68	18-69		
Median	47	49		
< 50	139 (56.7%	141 (57.6%)		
≥ 50	106 (43.3%)	104 (42.4%)		
WHO pathology			1.095	0.578
Type I	7 (2.9%)	4 (1.6%)		
Type II	10 (4.1%)	8 (3.3%)		
Type III	228 (93.0%)	233 (95.1%)		
ECOG performance status			1.783	0.182
0	221 (90.2%)	230 (93.9%)		
1	24 (9.8%)	15 (6.1%)		
T stage ^*^			2.736	0.434
T1	12 (4.9%)	11 (4.5%)		
T2	24 (9.8%)	34 (13.9%)		
T3	128 (52.2%)	114 (46.5%)		
T4	81 (33.1%)	86 (35.1%)		
N stage ^*^			2.532	0.470
N0	9 (3.7%)	6 (2.4%)		
N1	69 (28.2%)	58 (23.7%)		
N2	132 (53.9%)	148 (60.4%)		
N3	35 (14.2%)	33 (13.5%)		
Clinical stage ^*^			0.214	0.899
III	133 (54.3%)	132 (53.9%)		
IVA	77 (31.4%)	80 (32.7%)		
IVB	35 (14.3%)	33 (13.4%)		
IC cycle			2.107	0.147
2	142 (59.2%)	125 (51.0%)		
3-4	103 (40.8%)	120 (49.0%)		
AC			0.296	0.586
No	137 (55.9%)	130 (53.1%)		
Yes	108 (44.1%)	115 (46.9%)		

Abbreviations: WHO, World Health Organization; ECOG, Eastern Cooperative Oncology Group; IC, induction chemotherapy; AC, adjuvant chemotherapy; ^*^ The 7^th^ AJCC/UICC staging system.

### Survival

**Figure 1:**
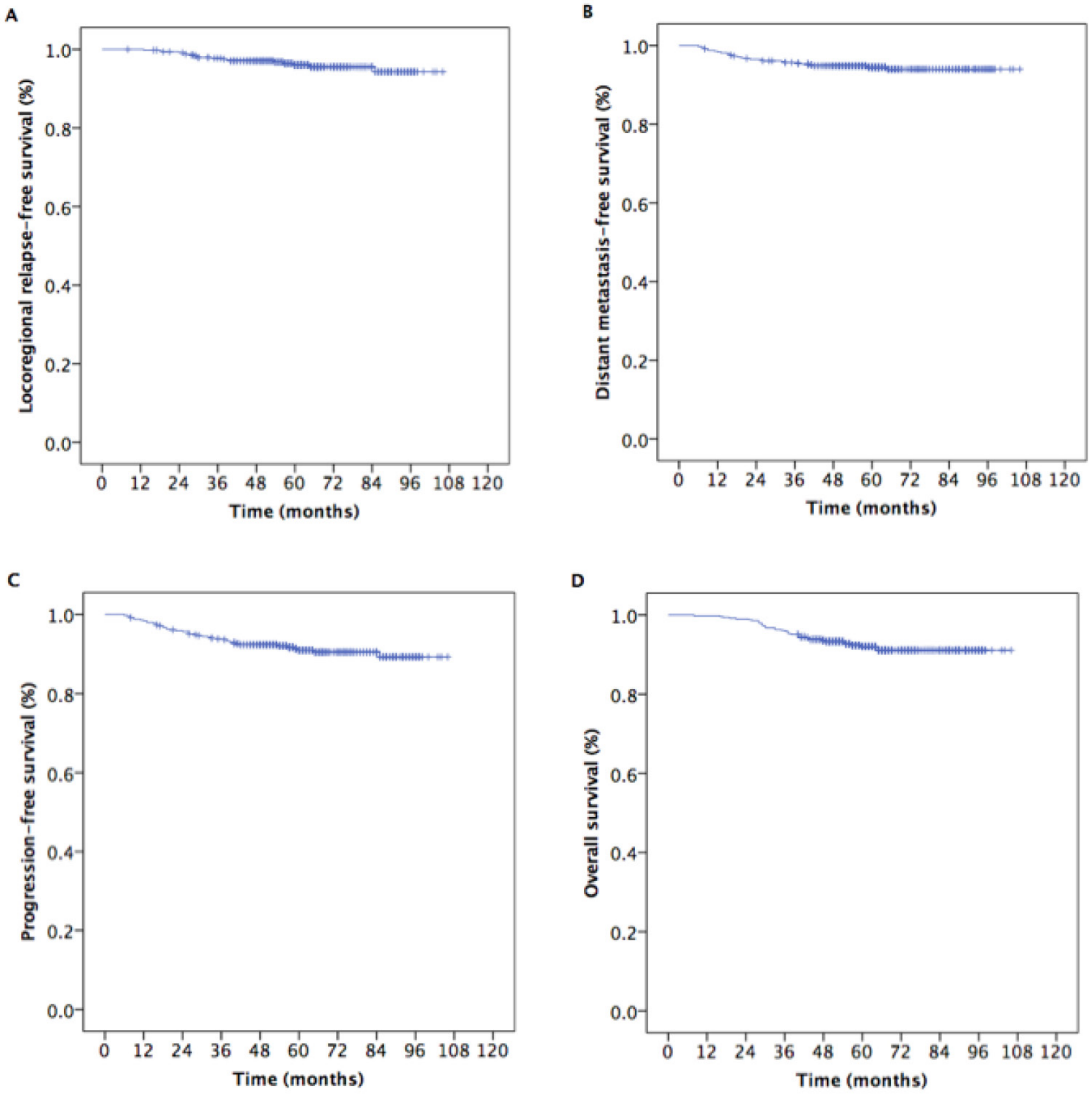
Kaplan-Meier estimates of survival in 490 patients with locoregionally advanced nasopharyngeal carcinoma

The follow-up rate for all locoregionally advanced NPC patients was 93.7%. At the median follow-up timepoint of 60 months (range, 8–106 months), the estimated 5-year locoregional relapse-free survival (LRRFS), distant metastasis-free survival (DMFS), progression-free survival (PFS), and overall survival (OS) rates were 96.0%, 94.5%, 91.0%, and 92.0%, respectively (Figure 1).

**Figure 2:**
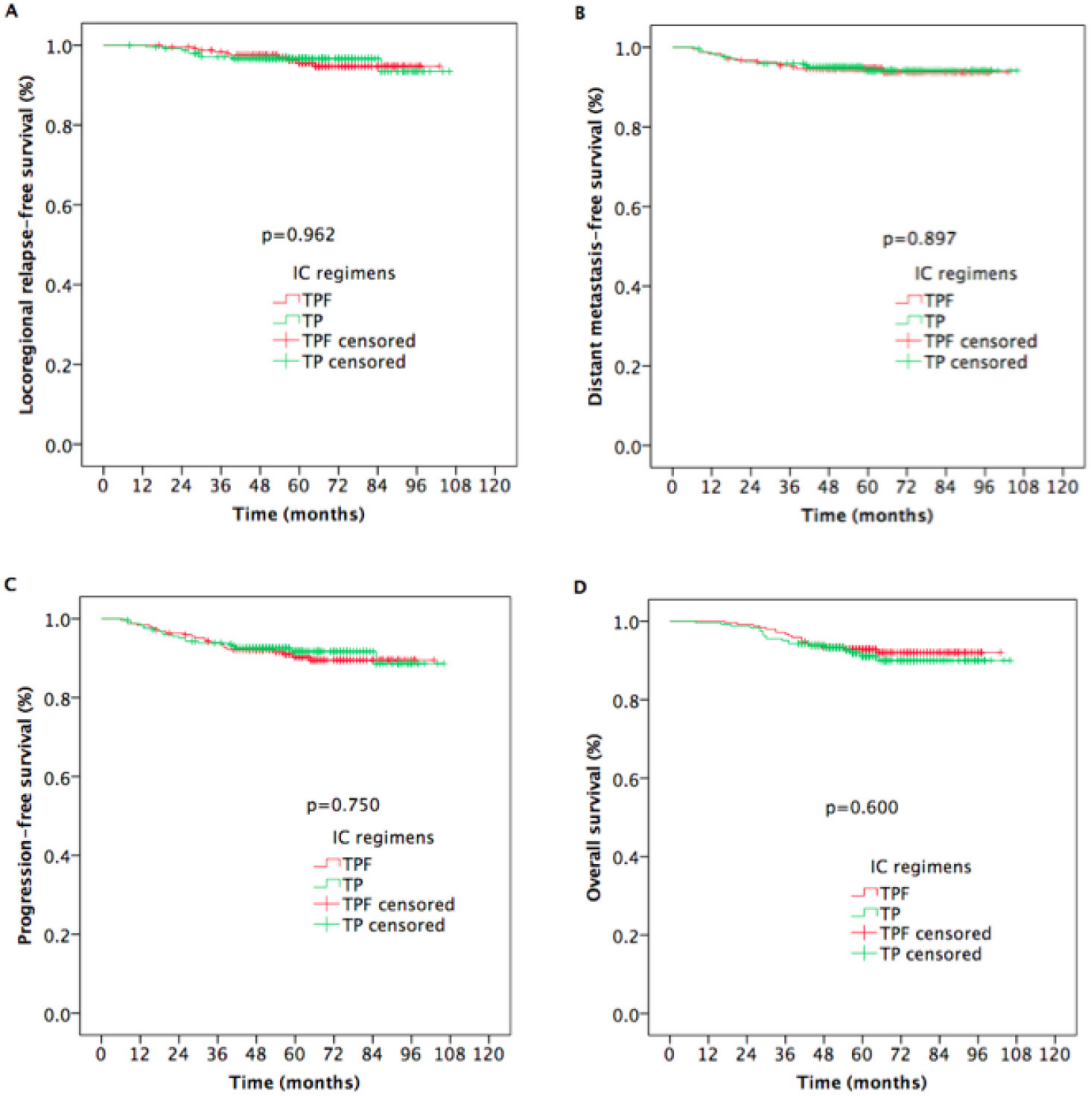
Kaplan-Meier estimates of survival outcomes in TPF and TP arm nasopharyngeal carcinoma patients

There were no statistically significant differences in 5-year LR-RFS, DMFS, PFS, or OS between the two arms (LRRFS: 95.6% vs. 96.7%, *p*=0.962, Figure 2A; DMFS: 94.7% vs. 94.2%, *p*=0.897, Figure 2B; PFS: 90.4% vs. 91.7%, *p*=0.750, Figure 2C; OS: 92.9% vs. 91.0%, *p*=0.600; Figure 2D).

### Patterns of treatment failure

Treatment failure occurred in 43 patients (8.8%) by the last follow-up. In the TPF arm, 23 patients (9.4%) experienced failure (locoregional relapse occurred in 9 patients, locoregional relapse and distant failure occurred in one patient, and distant metastases occurred in 13 patients). In the TP arm, 20 patients experienced failure (locoregional relapse occurred in 7 patients, locoregional relapse and distant failure occurred in 2 patients, and distant failure alone occurred in 11 patients). Patterns of treatment failure in NPC patients are summarized in Table 2. Median times to failure for the TPF and TP arms were 29 months (range, 6 to 65 months) and 19 months (range, 7 to 85 months), respectively.

**Table 2:** Patterns of treatment failure

Failure mode	TPF	TP	p
	N=245	N=245	
Locoregional	9	7	0.857
Locoregional and distant	1	2	
Distant	13	11	
No failure	222	225	

### Prognostic factors

Potential prognostic factors included patient age, gender, T category, N category, clinical stage, IC cycle, and IC regimen. We identified factors that influenced survival outcome and evaluated the prognostic role of these factors using univariate and multivariate analyses. Univariate analysis revealed that 5-year OS was better in patients younger than 50 than in those who were 50 or older (5-year OS: 95.3% vs. 87.5%, *p*=0.003), gender was associated with DMFS (5-year DMFS: 93.0% vs. 98.0%, *p*=0.028), and 5-year OS was worse in stage IVA/B patients than in stage III patients (5-year OS: 86.6% vs. 96.2%, *p*<0.001). The results of univariate analysis of the 490 locoregionally advanced NPC patients are shown in Table 3. Multivariate analysis indicated that age was an independent predictor of LRRFS (*p*=0.029). IC regimen was not an independent prognostic factor for any of the survival outcomes (Table 4).

**Table 3:** Prognostic factors in 490 NPC patients identified using univariate analysis

Characteristic	N	LRRFS (%)	p	DMFS (%)	p	PFS (%)	p	OS (%)	p
Age			0.050		0.340		0.761		0.003
< 50	280	96.8		93.3		90.5		95.3	
≥ 50	210	95.1		86.2		91.8		87.5	
Gender			0.517		0.028		0.175		0.360
Male	94	96.4		93.0		89.7		91.3	
Female	38	95.2		98.0		93.9		93.7	
T stage			0.306		0.820		0.497		0.867
T1-2	82	94.6		95.1		89.9		92.3	
T3-4	408	96.3		94.3		91.2		92.0	
N stage			0.788		0.211		0.373		0.136
N0-1	142	96.4		96.4		92.9		94.5	
N2-3	348	95.9		93.7		90.2		91.0	
Clinical stage			0.370		0.166		0.073		<0.001
III	279	96.6		96.1		93.1		96.2	
IVA/B	211	95.2		92.3		88.2		86.6	
IC cycle			0.302		0.120		0.520		0.811
2	270	95.3		95.9		92.0		91.3	
3-4	220	96.8		92.7		89.6		93.0	
IC regimen			0.199		0.554		0.835		0.434
TPF	57	96.4		87.7		86.0		94.7	
TP	75	90.3		91.9		85.2		92.0	
AC			0.101		0.767		0.255		0.552
No	267	97.4		94.8		92.3		93.0	
Yes	223	94.5		94.1		89.6		90.8	

Abbreviations: LRRFS, locoregional relapse-free survival; DMFS, distant metastases-free survival; PFS, progression-free survival; OS, overall survival; IC, induction chemotherapy; TP, docetaxel/cisplatin; TPF, docetaxel/cisplatin/fluorouracil.

**Table 4:** Summary of multivariate analyses of prognostic factors in 490 NPC patients

Endpoint	Factor	HR	95%CI	p
LRRFS	Age: <50 years vs. ≥50 years	0.345	0.133-0.896	0.029
	Gender: male vs. female	0.581	0.224-1.503	0.263
	T category: T1-2 vs. T3-4	1.760	0.586-5.291	0.314
	N category: N0-1 vs. N2-3	0.975	0.323-2.942	0.965
	AC: no vs. yes	0.356	0.110-1.152	0.085
	IC cycle: 2 vs. 3-4	1.068	0.311-3.661	0.917
	IC regimen: TPF vs. TP	1.413	0.511-3.903	0.505
DMFS	Age: <50 years vs. ≥50 years	1.473	0.654-3.313	0.350
	Gender: male vs. female	3.346	0.998-11.216	0.050
	T category: T1-2 vs. T3-4	1.065	0.379-2.994	0.905
	N category: N0-1 vs. N2-3	0.588	0.215-1.606	0.300
	AC: no vs. yes	0.677	0.252-1.814	0.437
	IC cycle: 2 vs. 3-4	0.387	0.148-1.016	0.054
	IC regimen: TPF vs. TP	0.947	0.349-2.572	0.915
PFS	Age: <50 years vs. ≥50 years	0.874	0.473-1.613	0.666
	Gender: male vs. female	1.499	0.713-3.155	0.286
	T category: T1-2 vs. T3-4	1.245	0.570-2.717	0.583
	N category: N0-1 vs. N2-3	0.262	0.362-1.601	0.473
	AC: no vs. yes	0.490	0.224-1.073	0.074
	IC cycle: 2 vs. 3-4	0.569	0.263-1.230	0.151
	IC regimen: TPF vs. TP	1.247	0.595-2.613	0.559
OS	Age: <50 years vs. ≥50 years	0.353	0.178-0.698	0.003
	Gender: male vs. female	1.156	0.542-2.468	0.707
	T category: T1-2 vs. T3-4	0.919	0.392-2.156	0.846
	N category: N0-1 vs. N2-3	0.506	0.217-1.182	0.115
	AC: no vs. yes	0.824	0.373-1.820	0.632
	IC cycle: 2 vs. 3-4	0.778	0.337-1.796	0.557
	IC regimen: TPF vs. TP	0.943	0.430-2.069	0.884

Abbreviations: LRRFS, locoregional relapse-free survival; DMFS, distant metastasis-free survival; PFS, progression-free survival; OS, overall survival; IC, induction chemotherapy; AC, adjuvant chemotherapy; TP, docetaxel/cisplatin; TPF, docetaxel/cisplatin/fluorouracil.

### Safety and toxicity

Hematologic and non-hematologic toxicities were the most common side effects observed after treatment. Incidences of acute toxicities resulting from IC and CCRT are listed in Table 5. Incidence of grade 3 or higher leukocytopenia and neutropenia was higher in TPF arm patients than in TP arm patients during IC treatment (58.4% vs. 20.4%, p<0.001; 66.1% vs. 22.4%, p<0.001). Additionally, more patients in the TPF arm suffered diarrhea than did those in the TP arm (33.1% vs. 7.8%, p=0.033). There were no other significant differences in toxicities between the two arms. In addition, no statistically significant differences in rates of hematologic and non-hematologic complications were observed between the two arms during CCRT.

**Table 5:** Toxicity from IC and CCRT between the two arms

Adverse event (toxicity grade)	IC					CCRT				
	TPF arm		TP arm		p	TPF arm		TP arm		p
	1-2	3-4	1-2	3-4		1-2	3-4	1-2	3-4	
Hematologic										
Leukocytopenia	99	143	172	50	<0.001	163	22	154	46	0.516
Neutropenia	60	162	145	55	<0.001	142	43	138	33	0502
Anemia	73	4	87	7	0.157	60	0	79	0	0.691
Thrombocytopenia	48	4	49	3	0.128	51	13	36	7	0.975
Liver function	154	4	105	2	0.520	47	0	43	0	0.055
Renal function	7	0	7	0	0.254	13	0	11	0	0.277
Non-hematologic										
Mucositis	30	0	25	0	0.874	232	13	232	13	1.0
Dermatitis	13	0	8	0	0.559	239	6	241	4	0.518
Diarrhea	77	4	19	0	0.033	22	4	17	2	0.770
Nausea/vomiting	47	4	35	4	0.937	34	1	33	3	0.099

Abbreviations: IC, induction chemotherapy; CCRT, concurrent chemoradiotherapy.

## DISCUSSION

Few studies have compared the efficacy and safety of TPF and TP in patients with locoregionally advanced NPC. To the best of our knowledge, this is the first study to compare long-term survival outcomes and toxicities of TPF and TP in a large sample size of locoregionally advanced NPC patients. We found that 5-year LRRFS (95.6% vs. 96.7%), DMFS (94.7% vs. 94.2%), PFS (90.4% vs. 91.7%), and OS (92.9% vs. 91%) rates did not differ between the two treatment arms. However, incidences of leucopenia, neutropenia, and diarrhea were lower in TP arm patients than in TPF arm patients. These results indicate that TP-based IC has similar efficacy to, but is associated with fewer grade 3/4 acute toxicities than, TPF treatment; the omission of 5-fluorouracil from IC therefore did not affect survival outcomes.

We also examined the prognostic value of various factors, including patient age, gender, T category, N Category, clinical stage, AC, IC cycle, and IC regimen. Gender was an independent prognostic factor of LRRFS, while age and clinical stage were independent predictors of OS; in contrast, IC regimen was not an independent prognostic factor for any of the survival outcome measures.

Survival improvements following the addition of docetaxel to IC with cisplatin and 5-fluorouracil were first described in locoregionally advanced head and neck cancer [17–19]. An IC regimen that included docetaxel also improved survival in patients with locoregionally advanced NPC [20–25]. In a randomized phase III study, 3 cycles of TPF IC followed by CCRT improved 3-year failure-free survival rates compared to CCRT alone (80% vs. 72%) [20]. Kong *et al*. reported that the addition of TPF IC to CCRT was associated with prolonged survival, with a 3-year LRFS, DMFS, PFS, and OS rates of 93.9%, 90.5%, 78.2%, and 94.8%, respectively, in locoregionally advanced NPC patients [21]. Kawahira *et. al*. showed that TPF IC before CCRT reduced distant metastasis in patients with nodal stage N2-3 disease [27]. However, Qu *et al*. found that the addition of TPF IC to CCRT did not improve 5-year OS (78.3% vs. 82.7%) or PFS (72.5% vs. 68.2%) [28].

Hassan *et al*. demonstrated that TP IC before CCRT provided good local control with an acceptable toxicity profile in locoregionally advanced NPC patients [22]. In a randomized phase II trial, Hui *et al*. observed that adding 2 cycles of TP IC to CCRT increased 3-year OS compared to CCRT alone (94.1% vs. 67.7%) [23]. In another phase II trial by Zhong *et al*., the addition of TP IC to CCRT resulted in 3-year OS and PFS rates of 94.1% and 72.7%, respectively [24].

While first-line IC regimens consisting of docetaxel plus cisplatin with or without 5-fluorouracil have resulted in excellent survival outcomes in locoregionally advanced NPC patients, few studies have compared the efficacy and toxicities of TP and TPF before CCRT in these patients. In our previous phase II study that examined the efficacy and tolerability of adding TPF or TP to concurrent chemotherapy and IMRT in locoregionally advanced NPC patients [25], both IC regimens resulted in similar survival outcomes, but the sample size was small and the follow-up time was short. We therefore conducted this observational study to investigate long-term survival outcomes in a large group of patients who received either TPF or TP. We observed that long-term survival outcomes were similar among patients in the TP and TPF arms, indicating that omission of 5-fluorouracil from TPF IC did not affect survival.

Factors that are often associated with prognostic value include age, gender, clinical stage, IC cycle, and IC regimen. Here, univariate analysis revealed that age, gender, and clinical stage impacted survival outcomes, while age and gender were identified as independent prognostic factors in multivariate analysis. However, neither univariate nor multivariate analysis indicated that IC regimen affected any of the survival outcomes. This result is particularly informative because the 490 patients examined in this study were paired in our analysis; thus, these findings strongly suggest that TPF and TP IC before CCRT result in similar survival outcomes.

Hematologic and non-hematologic toxicities were the most commonly observed side effects in patients during the treatment period. Incidences of grade 3 or higher leukocytopenia and neutropenia lower in patients who received TP than in those who received TPF (20.4% vs. 58.4% and 22.4% vs. 66.1%). The incidences of hematologic toxicities observed after TPF treatment in this study were similar to those in previous studies (ranging from 55-83%) [17, 18, 21, 29]. All patients in this study received prophylaxis leukocyte therapy in the form of recombinant granulocyte colony-stimulating factor (GCSF), which allowed those who experienced grade 3/4 leukocytopenia and neutropenia during IC treatment to continue with chemotherapy as initially scheduled. Non-hematologic side effects, such as mucositis, dermatitis, diarrhea, and nausea/vomiting, were mild to moderate. The incidence of diarrhea was lower in TP arm patients than in TPF arm patients (7.8% vs. 33.1%); incidences of the other complications did not differ between two arms.

Some limitations of this study should be considered when interpreting the results. First, this retrospective study was conducted at single center. Second, only acute treatment-associated toxicities were evaluated; later-stage complications were not examined. Third, acute toxicities were assessed based only on information provided in medical record. Finally, as in many retrospective studies, data was incomplete for many patients. Our results should therefore be regarded as preliminary, and additional prospective clinical trials in larger patient populations should be conducted to confirm these findings.

In conclusion, we found that the addition of either TP or TPF-based IC to IMRT with concurrent chemotherapy resulted in similar improvements in survival, including LRRFS, DMFS, PFS, and OS, in locoregionally advanced NPC patients, while TP IC was associated with lower incidences of grade 3/4 acute toxicities. Thus, omission of 5-fluorouracil from TPF-based IC did not affect survival outcomes. However, additional randomized, controlled, multicenter phase III clinical trials are needed to assess the efficacy and toxicity of TP IC regimens.

## MATERIALS AND METHODS

### Patients

The patients enrolled in this study were hospitalized in the Department of Radiation Oncology, Zhejiang Cancer Hospital between May 2008 and April 2014. Eligible patients met the following criteria: (i) histologically confirmed locoregionally advanced NPC, (ii) Eastern Cooperative Oncology Group performance status ≤ 1, (iii) completion of radical IMRT, (iv) received IC before CCRT with or without AC, and (v) no previous anti-cancer treatment. Ultimately, data from 490 of the 650 patients initially examined were included in the study.

The exclusion criteria were as follows: patients who were 70 years old or older; received RT, chemotherapy or surgery for tumors; had distant metastases before treatment; were pregnant; had a history of other malignancy; had severe comorbidities. This retrospective study was approved by the Medical Ethics Committee and the institutional reviewed board of Zhejiang Cancer Hospital. All the patients provided informed consent.

Of the 650 patients initially enrolled, 252 were treated with TP-based IC before CCRT and 398 were treated with TPF-based IC before CCRT. Patients in the two arms were paired using PSM based on gender, age, pathological type, T stage, N stage, clinical stage, ECOG, and IC cycle. 245 patient pairs were examined.

### Baseline examinations

Patients underwent pretreatment evaluations that included complete histories, physical examinations, hematology and biochemistry profiles, chest radiographs, sonography of the abdomen, bone scans, magnetic response images of the nasopharynx, and nasopharyngoscopies. All patients were staged according to the 2010 American Joint Committee on Cancer staging system. Tumor histology was classified per the World Health Organization classification.

### Intensity-modulated radiotherapy

All patients underwent radical IMRT with simultaneous integrated boost technique using 6 MV photons within 2-3 weeks after IC. Target volumes of NPC during IMRT treatment were delineated as described previously [25, 30–33]. Briefly, gross tumor volumes (GTV) of the primary tumor (GVTnx) and metastatic lymph nodes (GTVnd) were delineated according to pre- and post-IC MR images, respectively. The nasopharynx clinical target volume (CTVnx) was defined as GTVnx plus a 7-mm margin that encompassed the nasopharyngeal mucosa plus 5 mm submucosal volume. The high-risk clinical target volume (CTV1) included the entire nasopharyngeal cavity, the anterior one- to two-thirds of the clivus, the skull base, the pterygoid plates, the parapharyngeal space, the inferior sphenoid sinus, the posterior one-quarter to one-third of the nasal cavity, the maxillary sinus, and any lymph nodes in drainage pathways containing metastatic lymph nodes. The low-risk clinical target volume (CTV2) included levels IV and Vb without metastatic cervical lymph nodes.

The PTV was constructed automatically based on each volume with an additional 3-mm margin in three dimensions to account for set-up variability. None of the PTVs, including PGTVnx, PTVnx, PTV1, and PTV2, were delineated outside of the skin surface. Critical normal structures, including the brainstem, spinal cord, parotid glands, optic nerves, chiasm, lens, eyeballs, temporal lobes, temporomandibular joints, mandible, and hypophysis, were contoured and set as OARs during optimization.

The prescribed radiation dose was 69 or 72 Gy to PGTVnx, 66-70 Gy to PGTVnd, 62-66 Gy to PTVnx, 60-63 Gy to PTV1, and 51-54 Gy to PTV2, delivered in 30 or 33 fractions. Radiation was delivered once daily and at five fractions per week over 6 −6.5 weeks for IMRT planning. The dose to OAR was limited based on the RTOG 0225 protocol.

### Chemotherapy

All patients were given two to four cycles of platinum-based induction chemotherapy three times per week. TPF (docetaxel 60 mg/m^2^/day on day 1, cisplatin 25 mg/m^2^/day on days 1-3, and 5-fluorouracil 500 mg/m^2^/day on days 1-3) or TP (docetaxel 60 mg/m^2^/day on day 1, cisplatin 25 mg/m^2^/day on days 1-3) IC regimens were used.

The patients in this study also underwent concurrent chemotherapy with cisplatin (80 mg/m^2^) divided across 3 days and received adjuvant chemotherapy with FP (cisplatin 25 mg/m^2^/day and 5-fluorouracil 500 mg/m^2^/day on days 1–3) within 3-4 weeks after RT.

### Patient evaluation and follow-up

Tumor responses were assessed based on MRI and nasopharynx fiberscope according to the Response Evaluation Criteria for Solid Tumors on three occasions: after the completion of induction chemotherapy, at the end of IMRT, and 3 months after radiation. Adverse effects resulting from systemic chemotherapy were graded using the National Cancer Institute Common Toxicity Criteria (NCI CTCAE, version 3.0), whereas RT-induced toxicities were scored according to the Acute and Late Radiation Morbidity Scoring Criteria of the Radiation Therapy Oncology Group (RTOG).

All the subjects underwent weekly examinations for treatment response and toxicities during radiation therapy. Patient followed-ups were conducted every 3 months for the first 2 years, every 6 months from the third to the fifth year, and annually thereafter. Each follow-up included careful examination of the nasopharynx and neck nodes by an experienced doctor, MRI scan of the nasopharynx, nasopharynx fiberscope, chest computed tomography radiograph, and ultrasound of abdomen 3 months after the completion of RT and every 6–12 months thereafter. Additional examinations were performed when indicated to evaluate local relapse or distant metastasis.

### Statistical analysis

The endpoints examined included LRRFS, DMFS, PFS, OS, and acute toxicities after IC and CCRT. OS was calculated from the date of enrollment in the study to the date of death or the last follow-up. LRRFS, DMFS, and PFS were calculated from the date of enrollment to the date of locoregional relapse, occurrence of distant metastasis, and disease progression, respectively, or the date of the last follow-up. After recurrence or metastasis, patients received salvage therapy at the discretion of their physicians.

Descriptive statistics were used to compare patient characteristics, treatment adherence, and patterns of failure between the two arms. Independent sample non-parametric tests were used to compare acute toxicity between the two arms. Survival curves were generated using the Kaplan-Meier method and compared using log-rank tests. Multivariate analysis was performed using Cox regression models to identify significant prognostic factors. Hazard ratios (HRs) and 95% confidence intervals (CIs) were calculated for each prognostic factor. IBM SPSS Statistics version 19.0 was used for all data analysis, and *p* < 0.05 was considered statistically significant.
